# Technologies for studying phase-separated biomolecular condensates

**DOI:** 10.1007/s44307-024-00020-0

**Published:** 2024-03-07

**Authors:** Boyuan Deng, Gang Wan

**Affiliations:** Guangdong Provincial Key Laboratory of Pharmaceutical Functional Genes, MOE Key Laboratory of Gene Function and Regulation, State Key Laboratory of Biocontrol, School of Life Sciences, Sun Yat-Sen University, GuangZhou, GuangDong China

**Keywords:** Biomolecular condensates, Membrane-less organelles, Phase separation, Technologies

## Abstract

Biomolecular condensates, also referred to as membrane-less organelles, function as fundamental organizational units within cells. These structures primarily form through liquid–liquid phase separation, a process in which proteins and nucleic acids segregate from the surrounding milieu to assemble into micron-scale structures. By concentrating functionally related proteins and nucleic acids, these biomolecular condensates regulate a myriad of essential cellular processes. To study these significant and intricate organelles, a range of technologies have been either adapted or developed. In this review, we provide an overview of the most utilized technologies in this rapidly evolving field. These include methods used to identify new condensates, explore their components, investigate their properties and spatiotemporal regulation, and understand the organizational principles governing these condensates. We also discuss potential challenges and review current advancements in applying the principles of biomolecular condensates to the development of new technologies, such as those in synthetic biology.

## Introduction

Cells use compartmentalization to organize various biomolecules, including proteins, nucleic acids, lipids, and carbohydrates, to carry out biochemical reactions in an orderly and effective manner, thereby sustaining life. The traditional form of compartmentalization involves membrane-bound organelles in eukaryotic cells, such as the nucleus, lysosomes, and mitochondria. Over the past decade, membrane-less organelles, also known as biomolecular condensates, have increasingly been recognized as an essential form of compartmentalization. Examples of these include stress granules (Protter and Parker 2016), P bodies (Luo et al. 2018), nucleolus (Pederson 2011), transcriptional hubs (Boija, et al. 2018), heterochromatin (Larson et al. 2017), and germ granules (So et al. 2021), among many others. A growing number of biomolecular condensates are being discovered, and many more are yet to be identified, either through serendipitous findings or systematic screenings (Hou et al. 2023; Fong et al. 2013).

Although membrane-less organelles were known for a long time, it wasn't until the pivotal research on P granules in *Caenorhabditis elegans* that it was understood that these membrane-less organelles largely form through a process known as phase separation (Brangwynne et al. 2009). Historically, these non-membrane-bound compartments have been referred to by various names, including bodies, granules, speckles, puncta, and aggregates. More recently, biomolecular condensates have been proposed to encompass the characteristics of all these structures: concentrating molecules and comprising biological molecules (Banani et al. 2017). Phase separation is a well-established phenomenon in polymer chemistry and soft matter biophysics (Flory 1953). Essentially, biomolecular condensates are formed when proteins and nucleic acids separate from the surrounding cytoplasm or nucleoplasm. The primary driving force behind this phase separation is weak multivalent interactions (Li et al. 2012), which are typically mediated by intrinsically disordered regions (IDRs), RNA binding domains, and/or RNA molecules (Molliex et al. 2015). In some instances, IDRs and DNA molecules also contribute to this process (Du and Chen 2018). In addition, tandem structured domains can also drive phase separation through multivalent interactions (Li et al. 2012; Zeng, et al. 2016).

Biomolecular condensates typically consist of dozens, often hundreds, of protein and RNA components. Current research suggests that proteins or RNAs with a high capacity for phase separation act as scaffolds. Other proteins or RNAs, referred to as "clients", are recruited to biomolecular condensates through protein–protein or protein-RNA interactions with these scaffolds (Banani et al. 2016). Consequently, biomolecular condensates are a complex network of protein and RNA interactions (Yang, et al. 2020).

Biomolecular condensates generally exhibit properties akin to liquid droplets and adopt spherical shapes. However, recent studies have identified biomolecular condensates with properties resembling gels, glass, or solids, and these can form structures such as sheets or malformed structures (Ma and Mayr 2018; Yu, et al. 2021; Yu, et al. 2021). The properties of biomolecular condensates are closely linked to their physiological functions. For example, a more solid property is often associated with long-term storage or pathogenic state (Patel et al. 2015). Furthermore, biomolecular condensates are not homogeneous structures; they often have substructures. For instance, many condensates exhibit shell/surface-core structures (Jain et al. 2016; Wan et al. 2021; Folkmann et al. 2021; Zhou et al. 2021). Moreover, different biomolecular condensates can form higher-order assemblies (Wan, et al. 2018; Feric et al. 2016). For instance, when stress granules form, they often interact with P bodies (Moon et al. 2019; Tauber, et al. 2020; Sanders, et al. 2020). The nucleolus represents a multiphase composition of at least three different condensates (Feric et al. 2016; Shan et al. 2023).

While normal biomolecular condensates are critical for numerous physiological processes, aberrant biomolecular condensates have been linked to many diseases, such as cancer and neurodegenerative diseases (Bouchard, et al. 2018; Zhou, et al. 2022). With more insights into biomolecular condensates, the knowledge has begun to guide drug screen from biomolecular condensates perspective or synthetic biology (Mitrea et al. 2022; Lim and Clark 2023).

In this review, we summarize the prevalent technologies employed to identify new biomolecular condensates and the components within them. We delve into the methodologies employed to investigate the properties of these condensates, including their substructure, spatiotemporal regulation, and higher-order structure assembly. Furthermore, we discuss how the principles underlying biomolecular condensates are being integrated into the realm of synthetic biology technologies.

## Identification of novel biomolecular condensates

In the past, biomolecular condensates were often identified sporadically, either through serendipity or genetic screens. Visualized through fluorescence imaging, these condensates typically appear as granular foci, generally spherical in shape, although some instances of amorphous clusters have been observed (Kang, et al. 2022). Recently, advancements in technology have enabled the discovery of biomolecular condensates through large-scale screens or bioinformatic approaches.

### Enrichment of proteins with phase separation potential by biotinylated isoxazole precipitation

**Fig. 1:**
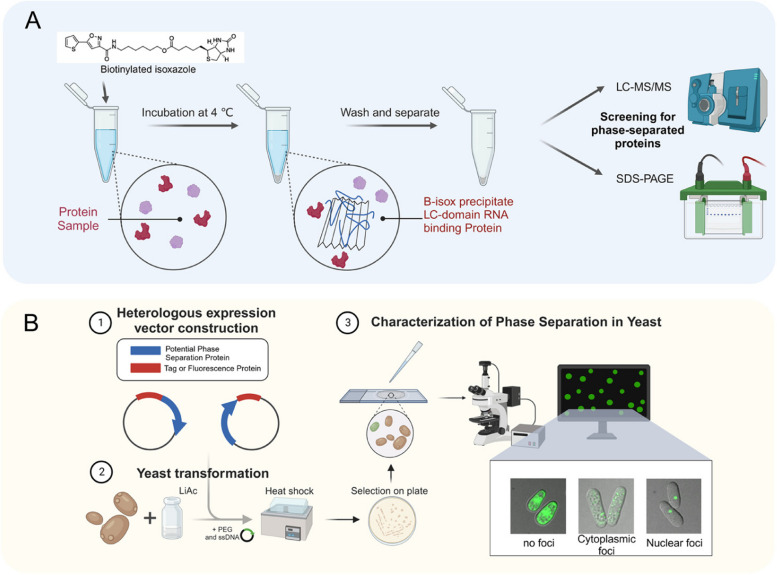
Screen for proteins with phase separation potential with biotinylated isoxazole (b-isox) enrichment, followed by their heterologous expression. **A** cell lysates are incubated with b-isox. The b-isox forms a curved surface, promoting binding with proteins possessing phase separation potential. These precipitated proteins can subsequently be identified using mass spectrometry. **B** proteins of interest are heterologous expressed, fused with a fluorescent protein such as GFP, in either human or yeast cells. The formation of cytoplasmic or nuclear foci serves as an indicator of these proteins' potential to form condensates

In a high-throughput drug screening, an isoxazole compound known as 5-aryl-isoxazole-3-carboxyamide was identified by researchers as a promoter of differentiation in mouse embryonic stem cells into cardiomyocytes (Sadek et al. 2008). To delve deeper into its cellular targets, a biotinylated derivative, referred to as biotinylated isoxazole (b-isox), was synthesized. B-isox demonstrated selective precipitation of proteins with the potential for phase separation, including translation initiation factors and proteins with KH, RRM, and DEAD box helicase domains. Importantly, it also precipitated various proteins linked with neurodegenerative diseases and mental retardation, such as FMR1, FUS, TDP43, and ataxin 2 (Kato et al. 2012). The microcrystal surface of b-isox appears to act as an organized template, stimulating the transformation of low-complexity sequences from a soluble state to polymerized, fiber-like structures. This characteristic implies that the surface facilitates the transition of disordered polypeptides into an extended β-strand state (Kato et al. 2012). B-isox precipitation, when combined with mass spectrometry, has been utilized to globally identify biomolecular condensates in various organisms, including mice (Kato et al. 2012), Drosophila (Kato et al. 2012), humans (Han et al. 2012) and Arabidopsis (Zhang et al. 2023a). Furthermore, the combination of b-isox precipitation and next-generation sequencing could provide a panoramic view of biomolecular condensates and their associated RNAs and DNAs (Han et al. 2012; Xing 2023) (Fig. 1A). However, as b-isox preferentially precipitates proteins with low-complexity sequences, it may not capture proteins that undergo phase separation via multivalent interactions between well-folded domains. Additionally, proteins that are either not expressed or expressed at considerably low levels in the input material may not be identified. Thus, input lysates from cellular fractionation or various developmental stages may be utilized to isolate more specific phase-separated proteins.

### Screen for proteins with the phase separation potential by expressing target proteins fused with fluorescent proteins

To identify proteins with potential for phase separation, these proteins can be tagged with fluorescent proteins and temporarily or stably expressed in a cell culture system or a heterogeneous yeast expression system (Fig. 1B). The formation of granular structures by these proteins can then be observed. For instance, a genome-wide screen was conducted to identify proteins localized to nuclear bodies. In this study, human open reading frames (ORFs) were sub-cloned to create fusion proteins with GFP, and these were transiently expressed in HeLa cells. This approach led to the identification of over 300 proteins with distinct nuclear body localizations, thus providing a valuable resource for future research (Fong et al. 2013).

Yeast serves as a potent genetic model system, offering substantial potential for screening proteins for phase separation. Previous studies have leveraged yeast models to investigate a variety of amyloid-associated disorders, including Huntington’s disease (Krobitsch and Lindquist 2000), Amyotrophic Lateral Sclerosis (Kryndushkin et al. 2011) and Alzheimer’s disease (Moosavi et al. 2015). This approach is particularly effective due to the presence of orthologues for approximately 20% of human disease-linked genes in S. cerevisiae, thereby enriching our understanding of disease mechanisms (Liu et al. 2017). In a fission yeast heterologous expression system, proteins enriched by b-isox in Arabidopsis were integrated into an endogenous locus. Remarkably, 63% of the expressed randomly selected RNA-binding proteins (RBPs) formed visible condensates in yeast cells (Zhang et al. 2023a). These works suggest that yeast can serve as a robust model for studying phase separation. The yeast expression system is a genetically tractable platform equipped with numerous established molecular tools. As a heterogeneous system with reduced interference, it can readily be applied to study the phase separation of proteins from various organisms, including mammals, Drosophila, *Caenorhabditis elegans*, and plants.

While heterogeneous expression serves as a rapid and robust method for screening proteins with phase separation potential, it's important to consider certain caveats. For instance, this approach often results in protein expression levels that exceed physiological concentrations, and phase separation is sensitive to a multitude of factors that are distinct from heterogeneous systems. Despite these considerations, heterogeneous expression can still offer a valuable resource for conducting more detailed investigations.

### Bioinformatics

Beyond experimental screening methods, a myriad of bioinformatics-based tools also plays a pivotal role in phase separation studies (Table 1). The wealth of experimental data serves as a valuable resource for bioinformatics, assisting in the identification of shared features and properties of proteins involved in phase separation. This characteristic is integral for the preliminary screening and analysis of a multitude of uncharacterized proteins.

**Table 1:** Phase separation related bioinformatics predictors and databases

Name	Publication Years	Main Features	Availability
PLAAC (Lancaster et al. 2014)	2014	Identify proteins with prion-like amino acid composition	http://plaac.wi.mit.edu/
catGranule (Bolognesi et al. 2016)	2016	Focus on structural disorder and nucleic acid binding propensities, sequence length and the presence of arginine, glycine, and phenylalanine	http://s.tartaglialab.com/
Pscore (Vernon 2018)	2018	Based on pi interaction frequency	/
LARK (Hughes et al. 2018)	2018	Low-complexity, aromatic-rich, kinked segments	/
Droppler (Raimondi et al. 2021)	2021	Neural network model is trained to predict, for each input protein sequence, its likelihood to undergo LLPS given a user-specified set of experimental conditions	https://bitbucket.org/grogdrinker/droppler/src/master/
PSAP (Mierlo et al. 2021)	2021	Introducing a machine-learning classifier designed to assess the phase separation likelihood of proteins within a proteome, relying solely on the amino acid content from a training set of known PPS proteins	https://github.com/Guido497/phase-separation
PSPredictor (Chu et al. 2022)	2022	A sequence-based machine learning PSP prediction tool	http://www.pkumdl.cn/PSPredictor
FuzDrop (Hatos et al. 2022)	2022	Based on a model in which the droplet state is stabilized by disordered interactions	https://fuzdrop.bio.unipd.it
DeePhase (Saar 2021)	2021	Combined engineering features from protein sequence and language model	https://deephase.ch.cam.ac.uk/
LLPhyscore (Cai et al. 2022)	2022	Based on a set of phase-separation-related physical interactions or features	https://github.com/julie-forman-kay-lab/LLPhyScore
CIDER (Holehouse et al. 2017)	2017	Based on Sequence-ensemble relationships of IDPs, include the patterns of long-range interactions, secondary structural preferences, and fluctuations about well-defined conformational elements	https://pappulab.wustl.edu/CIDERinfo.html
PONDR-FIT (Xue et al. 2010)	2010	Artificial neural network prediction method, with combining the outputs of several individual disorder predictors	www.disprot.org
ESpritz (Walsh et al. 2012)	2012	Based on neural networks and trained on three different flavors of disorder	http://protein.bio.unipd.it/espritz/
D2P2 (Oates et al. 2013)	2013	A repository of multiple structures and annotations based on genome-wide disorder predictors	https://d2p2.pro/
IUPred2A (Mészáros et al. 2018)	2018	Identify disordered protein regions using IUPred2 and disordered binding regions using ANCHOR2 and protein regions that do or do not adopt a stable structure depending on the redox state of their environment	https://iupred2a.elte.hu/plot_new
PhaSepDB (You et al. 2020)	2019	A manually curated database of phase separation associated proteins	http://db.phasep.pro/
PhaSePro (Mészáros et al. 2020)	2020	A manually curated database comprising experimentally validated LLPS driver proteins and protein regions	https://phasepro.elte.hu
LLPSDB (Wang, et al. 2022; Li et al. 2020b)	2022	A web-accessible database offers a comprehensive, curated collection of proteins involved in liquid–liquid phase separation, along with the corresponding experimental conditions in vitro, compiled from published literature	http://bio-comp.org.cn/llpsdbv2

Key features associated with the physical properties of amino acids, such as charged residues, aromatic rings, and the hydrophobic or hydrophilic nature, as well as the capacity to form hydrogen or disulfide bonds, play a crucial role in protein phase separation (Sun et al. 2022; Das et al. 2020; Schuster et al. 2020). Several predictors, grounded on the above-mentioned characteristics, can pinpoint proteins that undergo phase separation. Although phase separation is not reliant on a single type of protein interaction, early prediction methodologies primarily concentrated on a narrow spectrum of interaction types or sequence features. It's noteworthy that the multivalency of folded domains and oligomerization motifs is yet to be fully encapsulated to improve prediction accuracy (Xue et al. 2010).

As the body of experimental evidence regarding protein phase separation behavior expands, databases that compile a wide range of information have been established. These databases include data on protein phase separation behavior, protein sequence characteristics, and the physiological conditions under which in vitro phase separation occurs (Wang, et al. 2022; You et al. 2020). Second-generation phase separation prediction tools, like FuzDrop (Hardenberg et al. 2020), PSPredictor (Chu et al. 2022), and LLPhyScore (Cai et al. 2022), leverage extensive experimental data and utilize language models and machine learning. Machine learning has also been applied to identify granular structures in the Human Cell Atlas, generating a valuable resource for further exploration (Yu et al. 2021).

However, many current predictors do not account for post-translational modifications (PTMs), which limits their ability to analyze the regulatory effects of PTMs. Future prediction tools will likely need to incorporate more comprehensive data and employ advanced modeling techniques to address these limitations.

## Identification of components of biomolecular condensates

### Immunoprecipitation and FACS sorting

**Fig. 2:**
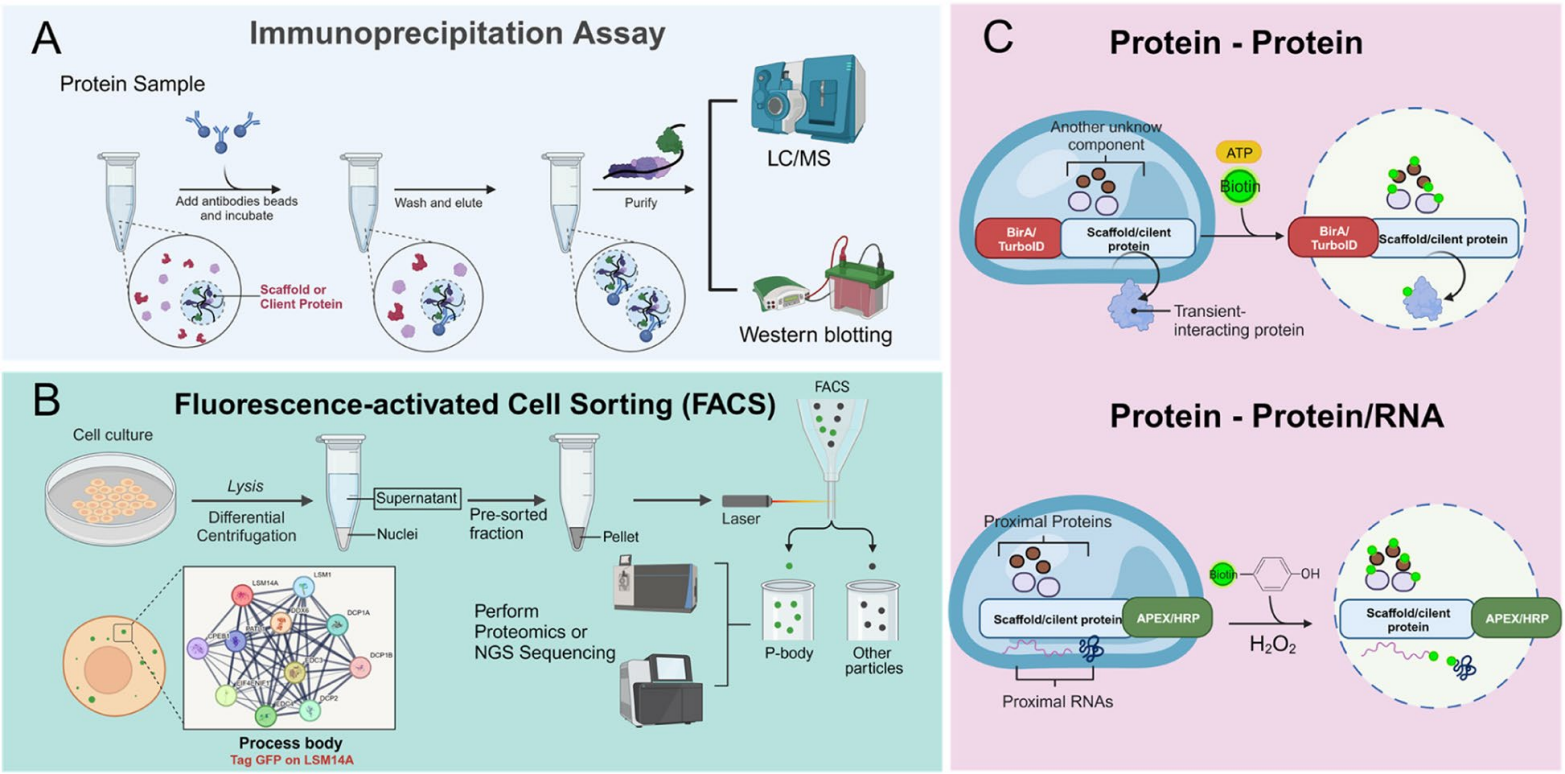
Approaches to study components of biomolecular condensates. **A**-**B** Condensates can be purified using either immunoprecipitation or fluorescence-activated cell sorting (FACS). Subsequent identification of protein and RNA constituents can be accomplished through mass spectrometry or RNA-sequencing (RNA-seq). **C** Alternatively, the presence of protein and RNA components within condensates can be elucidated using proximity biotin labeling techniques, such as TurboID or APEX2

Biomolecular condensates are complex structures composed of hundreds of protein and RNA components. Broadly, these components can be categorized into two groups: scaffolds and clients. Scaffolds are a few proteins or RNAs with a high propensity for phase separation, and their loss can significantly impact the integrity of the condensates. On the other hand, clients are proteins or RNAs that are recruited into the condensates through either direct or indirect interactions with the scaffolds. While clients may not be essential for the structural formation of the condensates, they can influence their properties (Ditlev et al. 2018). Biomolecular condensates can be isolated using techniques such as immunoprecipitation, which targets the scaffold proteins (Jain et al. 2016), or fluorescence-activated cell sorting (FACS) to sort fluorescently labeled condensates (Hubstenberger, et al. 2017) (Fig. 2A-B). Once purified, these biomolecular condensates can be subjected to mass spectrometry and RNA-sequencing for further analysis. However, an important caveat to these approaches is that due to the liquid nature of the condensates and weak and transient interaction of clients with scaffold, it's possible that only the core part of the biomolecular condensates is purified (Jain et al. 2016). This could result in a significant loss of information about the full composition and structure of the condensates. Meanwhile, only a few well-studied biomolecular condensates have been purified using these approaches, so they may not be applicable to the vast majority of biomolecular condensates.

### Proximity biotin labeling

Biomolecular condensates, which are composed of scaffolds and client components that assemble through weak or transient interactions, present challenges for traditional immunoprecipitation methods due to the difficulty in capturing these fleeting interactions. In contrast, proximity biotin labeling technology, an approach that effectively marks proteins or RNAs in the vicinity of a target protein, serves as a valuable supplement to affinity purification for capturing transient cellular interactions.

This technology is particularly useful in studying biomolecular condensates. The process encompasses the labeling of neighboring molecules using biotin ligase enzymes, such as BioID (Roux et al. 2012), TurboID (label proteins within 20 nm radium) (Branon et al. 2018), and APEX2 (label proteins and RNAs within 20 nm radium) (Li et al. 2020a). These modified enzymes, when fused with the protein within condensates, enable promiscuous biotin labeling of condensates components (Fig. 2C). For instance, in the study of PML nuclear bodies, which recruit different client proteins depending on the cellular context, researchers engineered a cell line expressing a GFP-PML-TurboID fusion protein. This allowed the identification of TRIM33, a client protein that influences PML-NBs localization and functions as a chromatin reader, through biotinylation and streptavidin purification. This discovery suggests that PML-NBs can recruit a diverse range of client proteins in a cell-context-dependent manner (Sun et al. 2023). Furthermore, APEX2 mediated biotinylation has facilitated the identification of variations in stress granule composition under stress conditions and across different cell types (Cui, et al. 2023; Markmiller et al. 2018).

While proximity labeling technology offers a robust approach to studying biomolecular condensates, it is not devoid of challenges. For instance, APEX labeling requires catalysis by H_2_O_2_, which poses significant biological toxicity risks for in vivo applications (Mathew et al. 2022). TurboID, while providing the highest biotin labeling activity to date, also suffers from high background labeling due to the use of endogenous biotin substrates. Additionally, the fusion of these enzymes to target proteins may compromise the integrity and function of the condensates. As such, optimizing experimental parameters is crucial for the precise identification of components within condensates. This includes implementing proper controls and adopting techniques like Split-TurboID. This method involves two inactive TurboID fragments that become active through specific interactions, offering enhanced targeting specificity compared to full-length enzymes (Cho et al. 2020). Despite these challenges, the potential of proximity labeling technology in unraveling the complex interactions within biomolecular condensates remains promising, provided that careful experimental design and methodological refinements are continuously pursued.

## Study the properties and assembly principle of biomolecular condensates

### In vitro reconstitution

A thorough examination of the properties of biomolecular condensates is crucial for grasping their biological roles. A key step in this exploration involves the in vitro reconstitution of simplified condensates of essential components, aimed at assessing their capacity for phase separation.

The process of in vitro reconstitution commences with the expression and purification of the target molecule. For proteins, this step involves the selection of appropriate expression systems (such as bacterial and baculovirus), tags (like His, MBP, or SUMO), and purification methodologies, all of which can impact phase separation properties. RNA molecules, on the other hand, are typically synthesized via in vitro transcription. The goal is often to obtain a significant amount of pure, tag-free protein that retains its biological activity. It's also crucial to consider how various fluorescent tags on proteins might influence their phase separation capabilities (Pandey et al. 2023).

Subsequently, the focus shifts to assembly events involving the key components. Most protein phase separations necessitate a molecular driving force, such as salt ions, ATP, DNA or RNA (Du and Chen 2018; Maharana et al. 2018; Shi et al. 2022). The presence of droplets indicates phase separation, which can be detected through methods such as solution turbidity, differential interference contrast microscopy, or fluorescence imaging microscopy (Molliex et al. 2015).

**Fig. 3:**
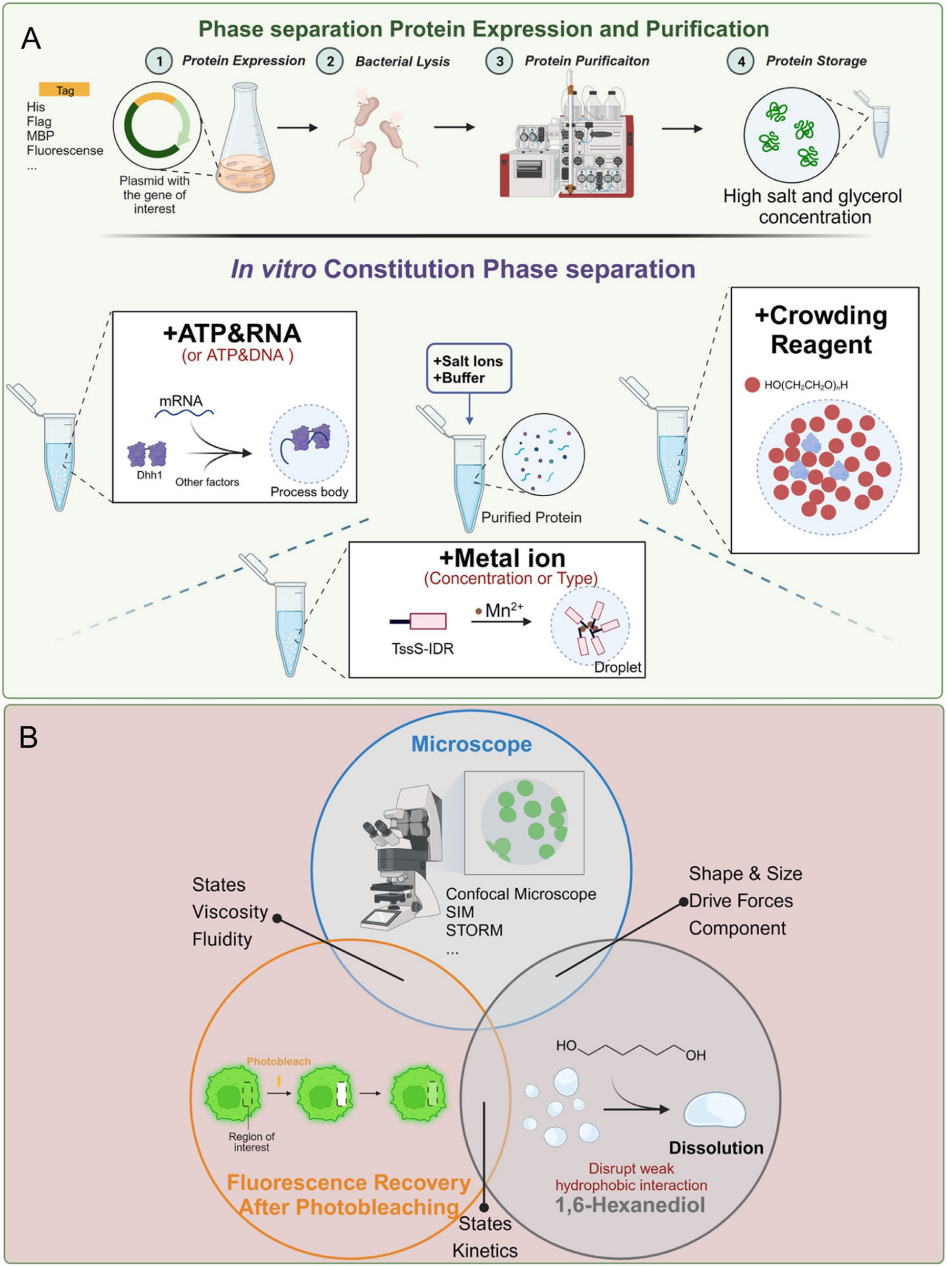
Technologies to study the property of condensates in vivo and in vitro. **A** Scaffold proteins within condensates can be expressed and subsequently purified. In vitro phase separation can be conducted using various protein concentrations, in combination with a range of RNA/DNA and salt concentrations. Crowding reagents can be utilized to simulate the crowded cellular environment, although this should be done with care. Each rectangle contains an example of recently published cases (Wang et al. 2023; Wang et al. 2022; Linsenmeier et al. 2022). **B** The properties of biomolecular condensates, such as sub-structure, viscosity, and spatiotemporal distribution, can be investigated using imaging approaches, FRAP, 1,6-Hexanediol treatment, either in vivo or in vitro

A phase diagram can be drawn after performing in vitro phase separation experiments with various concentrations of proteins, salts and nucleic acids. Crowding reagents such as PEG, Dextran and Ficoll are used to mimic crowding cellular environment. These three agents, available in a range of molecular weights, exhibit distinct structures and viscosities. For instance, PEG features a linear structure and lower viscosity compared to dextran and Ficoll. Conversely, Ficoll boasts a highly branched structure and higher viscosity in comparison to PEG and dextran. However, since they differ significantly from most cellular components, caution should be taken when interpreting phase separation results obtained using these crowding reagents (Tyrrell et al. 2015; Andre and Spruijt 2020). Condensates formed in vitro can replicate key characteristics observed in more complex condensates in vivo, including their states (liquid, gel, and solid), viscosity and driving force (Boeynaems et al. 2019; Niu et al. 2023). Post-translational modifications such as phosphorylation and methylation play crucial roles in protein phase separation (Qamar et al. 2018; Kim et al. 2019). By mimicking these modifications in vitro or reconstituting purified proteins with key residues for post-translational modifications mutated, researchers can study how they affect phase separation. Understanding these aspects is pivotal in deciphering the role of phase separation in biological processes (Patel et al. 2015; Mugler 2016; Elbaum-Garfinkle et al. 2015) (Fig. 3A).

However, in vitro reconstruction experiments have limitations. Often, these experiments involve protein or RNA concentrations that exceed physiological levels, potentially not providing an accurate representation of the dynamic physiological environment within cells. The physiological levels of proteins can be estimated by comparing them with proteins of known concentrations using quantitative Western blotting or fluorescence techniques (Bandaria et al. 2016; Jack, et al. 2022; Zhang et al. 2022). Whenever possible, in vitro reconstitution experiments are performed at physiologically relevant concentrations. Furthermore, due to the simplified nature of the components in in vitro reconstitution, interactions with other proteins may be missed. For instance, in the study of the COPII complex, the outer coat component Sec13/31 could not independently form a phase in vitro. However, upon the addition of the inner coat components Sec23/24, the condensed inner COPII coats recruited the outer coats to form liquid droplets, a process subsequently regulated by the latter (Wang et al. 2023).

### In vivo reconstitution

Given the complexity of mimicking the dynamic intracellular environment in vitro, researchers are increasingly adopting engineered phase-separating systems in vivo. These engineered platforms offer programmability and modularity, providing precise control over phase behavior within cellular contexts. While self-associating IDRs have been demonstrated to be necessary and sufficient for phase separation, additional domains such as RNA binding domains can further promote phase separation through multivalent interactions with RNA (Lin et al. 2015; Burke et al. 2015). Inspired by these findings, a key strategy known as optoDroplet has been developed. It utilizes light-induced phase separation to study domains required for phase separation and to spatiotemporally control phase separation in living cells (Shin, et al. 2017). Proteins like CRY2, capable of oligomerization or dimerization, can trigger the formation or dissociation of condensates under blue light (Kennedy et al. 2010). Fusing these photosensitive proteins with the IDRs of a target protein enhances the valency of the IDRs, enabling controlled condensate formation and dissolution (Shin, et al. 2017). In vivo reconstitution of phase separation facilitates the exploration of regulatory mechanisms in cellular processes, including reaction specificity, thermodynamics, and signaling pathways (Day et al. 2021). It also allows systematic investigation of the physical forces governing intracellular phase separation. CasDrop system, based on CRISPR/Cas9 and optogenetic technology, artificially controls liquid condensation at specific genomic loci. This technology demonstrates that nuclear condensates, such as those formed by transcriptional regulators, tend to nucleate and grow, physically excluding chromatin (Shin, et al. 2018).

However, empirical evidence suggests that in vivo reconstitution based on light control may not always align well with in vitro experiments. The propensity for cells to enter a state of supersaturation during periods of intense self-association may transform liquid-like condensates into gel-like or irreversibly aggregated states, potentially introducing toxicity (Shin, et al. 2017). Furthermore, achieving more precise and stable control of phase separation in vivo remains crucial goals. Quantifying multivalent states, elucidating interaction strengths, and constructing multilayer condensates represent envisioned directions for the future advancement of in vivo reconstruction phase separation technology.

### Study the properties and behavior of biomolecular condensates

The dynamics of biomolecular condensates are integral to their function. Most of these condensates resemble liquids, with biomolecules constantly moving within them and exchanging with the surrounding milieu. However, these condensates can exhibit a range of dynamics, even transitioning from one state to another. Their dynamics can span from liquid-like to gel-like, solid-like, or even amyloid-like. For instance, the first biomolecular condensate described as liquid-like was P granules in *Caenorhabditis elegans* (Brangwynne et al. 2009). The pericentriolar material (PCM) is a gel-like scaffold located near the centriole (Woodruff, et al. 2017), while Balbiani bodies display amyloid-like properties and form a stable matrix (Boke et al. 2016). In general, less dynamic biomolecular condensates are associated with critical cellular structures or long-term storage (Bose et al. 2022; Munder 2016). Conversely, liquid-like biomolecular condensates play numerous roles in rapid cellular processes or responses to external environmental cues (Watanabe 2021). To measure the dynamics of biomolecular condensates, fluorescence recovery after photobleaching (FRAP) is a frequently used technique for both in vitro constituted condensates and in vivo studies (Mitrea et al. 2018). In this method, the fluorescence of a small condensate area is bleached by a laser, and the fluorescence recovery of the same area is monitored over time (from seconds to minutes). To compensate for fluorescence bleaching during imaging, unbleached condensates are also imaged for comparison. A fast recovery rate (a few seconds) usually indicates a liquid-like property, while a recovery time of minutes or no recovery at all suggests a gel or solid-like state (Fig. 3B). It's important to note that the state of in vitro and in vivo condensates may not always align. In vitro reconstituted condensates may transition from a liquid-like to a solid-like state, a process known as aging.

When possible, the fusion behavior and fusion rate of condensates should be monitored, as these are strong indicators of a liquid-like state. Wetting behavior can also be observed as the shape of liquid condensates will be more spread when contact with membrane surface (Mangiarotti et al. 2023). Imaging and quantifying the shape of condensates can also reveal their dynamics (Baggett et al. 2022). A spherical shape is often, but not always, associated with a liquid-like property. Amorphous shapes, aggregates, or mesh-like structures may indicate a solid or amyloid-like state.

1,6-Hexanediol (1,6-HD) is an aliphatic alcohol widely used in studying the properties of biomolecular condensates. It possesses the ability to dissolve a broad array of these condensates, both in vivo and in vitro. It is hypothesized that 1,6-HD inhibits the phase separation of scaffold proteins by disrupting the weak hydrophobic protein–protein or protein-RNA interactions necessary for the formation of these condensates. With meticulous consideration of treatment duration, concentration, and controls, 1,6-HD has been instrumental in exploring the role of biomolecular condensates in 3D chromatin organization (Liu 2021). Therefore, when used judiciously, 1,6-HD may provide an approach to connect the loss of biomolecular condensate structure to biological function. To ensure reliable results, it's important to implement appropriate controls, such as using 2,5-hexanediol (2,5-HD) in parallel. It's also crucial to optimize a concentration range of 1,6-HD (1%-10%) and minimize treatment time to prevent cell apoptosis and secondary effects (Fig. 3B).

The morphological transformation, assembly, and disassembly of biomolecular condensates within a spatiotemporal context are of significant importance for understanding the physiological functions of these condensates. They may form in response to environmental cues such as water potential, temperature, osmotic pressure, or oxidative stress (Zhu et al. 2022; Majumder and Jain 2020; Ikeda et al. 2023; Watson et al. 2023; Riback, et al. 2017). Others may form in reaction to cellular density or mechanical signals (Zhang, et al. 2020). Certain condensates form temporarily during circadian rhythms (Zhuang, et al. 2023), cell cycles (Yamazaki et al. 2022), or developmental stages (Wan, et al. 2018). Therefore, tracking the behavior of these condensates within their spatiotemporal context provides valuable insights (Fig. 3B).

### Dissect the grammar of phase separation of scaffold proteins in biomolecular condensates

Scaffold proteins are integral to the integrity of condensates, making it crucial to dissect the molecular grammar of their phase separation. With detailed information about this molecular grammar, it becomes possible to link condensate structure with physiological roles. By examining the assembly status of client proteins following the depletion of candidate scaffold proteins, it's feasible to identify one or more key scaffold proteins. Scaffold proteins are characterized by having repeat modular domains or IDRs (Li et al. 2012; Kato et al. 2012). These domains can promote phase separation through weak multivalent interactions. The driving forces of weak multivalent interactions include π-cation interactions between aromatic amino acids and positively charged amino acids, electrostatic interactions between positively and negatively charged amino acids, and hydrophobic interactions. In theory, three or more such interactions can promote phase separation. To study the driving forces of scaffold proteins, a series of truncation mutations can be made to narrow down the domains critical for phase separation. These can be tested with in vivo and in vitro techniques, as previously described. Comparing full-length scaffold proteins, those with critical domains for phase separation removed, and those with only the critical domain for phase separation can provide valuable insight into their phase separation capacity and physiological roles (Alberti et al. 2018).

Once the critical domains required for phase separation are identified, a series of point mutations for key amino acids can be generated to further dissect the molecular mechanisms (Hondele et al. 2019). The amino acid composition in IDRs is typically biased, featuring an enrichment of aromatic, charged, and polar amino acids, including tyrosine, arginine, serine, glutamine, and glycine (Wang, et al. 2018). Grafting known domains with high phase separation capacity, such as FUS IDR, to replace the identified IDR can be very helpful (Qamar et al. 2018). Additionally, when feasible, exploring genetic disease mutation databases and linking disease mutations to phase separation behavior can directly associate phase separation with genetic diseases, thereby increasing clinical relevance (Banani et al. 2022).

### Study the higher organization of biomolecular condensates

Growing evidence suggests that biomolecular condensates are not homogeneous structures; rather, they often possess substructures, much like conventional membrane-bound organelles. Moreover, different condensates may form multiphase higher-order structures, or establish contact with other condensates or even membrane-bound organelles (Liao, et al. 2019). The immiscibility of multiphase condensate structures arises from differences in the biophysical properties of the condensates, such as surface tension, which is determined by sequence-encoded features of their macromolecular components (Feric et al. 2016). These organizations carry significant implications and functional relevance.

The higher organization of biomolecular condensates is beginning to be understood, but much work remains to dissect the mechanisms of organization and their functional significance. Biomolecular condensates are typically several hundred nanometers in size, a range close to the diffraction limit of microscopy (approximately 200 nm). As such, their substructures may be overlooked when observed with conventional confocal microscopy. Super-resolution microscopy techniques, such as SIM, STED, or STORM, which can reach resolutions of dozens of nanometers, can reveal these substructures and higher-order structures (Zhang et al. 2023c). Additionally, these structures may be reconstituted in vitro with key scaffold proteins, allowing for a detailed dissection of the mechanisms of structure formation (Mitrea et al. 2018).

Recent studies suggest that biomolecular condensates are complex protein–protein interaction networks, built on core node proteins and expanded by secondary node proteins (Yang, et al. 2020). The higher organization of different condensates is regulated by bridge proteins that connect nodes in different condensates. Proteins that reduce the valency of nodes, known as caps, also play a role in higher-order regulation (Sanders, et al. 2020). Therefore, the identification of nodes, bridges, and cap proteins may be key to understanding the higher organization architecture of condensates. A series of co-immunoprecipitation experiments or fluorescence resonance energy transfer (FRET) can be used to confirm protein–protein interactions within condensates and measure the interaction strength of query proteins (Gao et al. 2022).

## Application of biomolecular condensates principles in biotechnology

Biomolecular condensates serve as a means to enrich biomolecules. With the principles uncovered through intensive studies, it is now possible to apply this knowledge to the development of new technologies, particularly in the field of synthetic biology (Lim and Clark 2023). In this context, we provide an overview of several notable examples.

### Enhancing CRISPR based gene activation

**Fig. 4:**
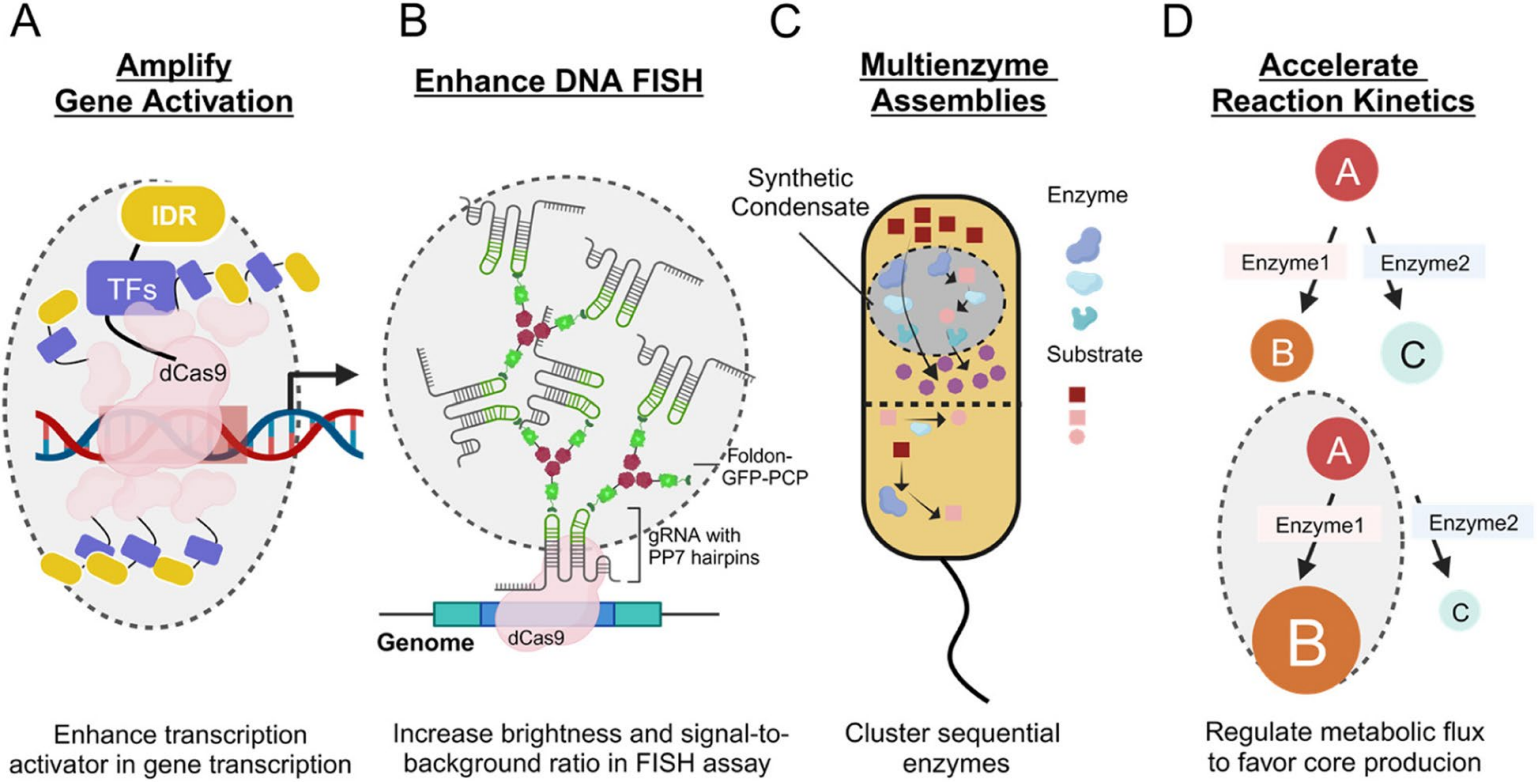
Applications of the principles of biomolecular condensates in biotechnology. Synthetic condensates can serve multiple purposes. Synthetic condensates can be employed to enrich transcription activators. When guided by dCas9, these condensates can enhance gene activation, exhibiting superior performance compared to the use of transcription activators alone (**A**). Synthetic condensates can also be harnessed to amplify DNA FISH signals by improving the signal-to-background ratio (**B**). Furthermore, they can concentrate single or multiple enzymes, thereby accelerating biochemical reactions (**C**-**D**)

The dCas9 protein, when mutated at catalytic residues, can guide fused proteins to specific genomic sites. By fusing the transcription activator VP64 to dCas9, the transcription of target genes can be activated (Liu et al. 2023). To amplify gene activation, an approach involving synthetic condensates has been adopted. Essentially, IDRs from NUP98 or FUS/DDX4/TAF15 are fused with VP64. This design leads to the clustering of VP64 within condensates, significantly enhancing gene activation levels by several to dozens of times (Liu, et al. 2023; Ma et al. 2023) (Fig. 4A).

### Enhanced DNA FISH

Biomolecular condensates can be utilized to amplify signals and improve the signal-to-noise ratio. Visualizing specific, especially non-repetitive, DNA sequences within the genome presents a significant challenge. In recent years, dCas9/sgRNA-guided DNA Fluorescence in Situ Hybridization (FISH) techniques have been developed (Wang et al. 2019), but these often suffer from high background noise. To address this issue, a technique known as CRISPR FISHer has been developed, which amplifies DNA FISH signals through phase separation. Essentially, a Foldon trimerization module is fused to GFP-PCP, where PCP can recognize two or more PP7 stem-loops designed into the sgRNAs. This design fosters multivalent interactions, promoting phase separation and forming condensates that enrich Foldon-GFP-PCP molecules (Lyu et al. 2022). When this module is fused with dCas9/sgRNAs, a single gene locus can be detected (Fig. 4B).

### Synthetic biomolecular condensates for synthetic biology

Synthetic biology employs a bottom-up paradigm for the purposeful design and construction of novel biological components and systems. Biomolecular condensates play a crucial role by dynamically forming compartments within cells. These condensates facilitate site-specific biochemical reactions and signal transduction at the subcellular level (Alghoul, et al. 2023). In the field of microbial metabolic regulation, conventional methods like overexpressing or deleting specific metabolic nodes often present challenges. A strategy involves heterologous expression of the RGG domain in the P granule protein LAF-1, leading to the formation of intracellular condensates (Elbaum-Garfinkle et al. 2015). This artificial condensate enables multienzyme assemblies, as demonstrated by the fusion expression of key metabolic enzymes like GDP-L-fucose, crucial in fucosyllactose synthesis. Such synthetic condensates, by clustering sequential enzymes, have shown efficacy in improving the titer and yield of targeted products (Wan et al. 2023) (Fig. 4C-D).

Additional control over the catalytic compartments can be achieved by engineering their formation and/or dissolution to be responsive to external triggers. As the phase separation is mediated by multivalent protein–protein interactions, the introduction of stimuli-responsive interaction domains can facilitate the conditional formation of biomolecular condensates. A condensate-based system has been designed to optogenetically control the metabolic flux of the deoxyviolacein biosynthesis pathway. The N-terminal disordered region of the Fused in Sarcoma (FUSN) protein was fused with the VioC/E enzymes, as well as the Cry2 domain, which undergoes light-inducible oligomerization (Zhao et al. 2019). This innovative approach enhances product yields. Such strategies, employing synthetic condensates, exemplify the precision and efficiency that synthetic condensates bring to cellular processes, highlighting their transformative potential across diverse fields and future advancements.

## Concluding remarks

In this review, we have summarized the most used techniques in the field of biomolecular condensates. We have provided a concise summary of the key technologies and compared their respective advantages and shortcomings in Tables 2 and 3. However, our understanding of these condensates is far from complete, and there is a pressing need for new techniques especially interdisciplinary approaches to gain more critical insights. Firstly, while much work has been done at the cellular level, further studies should be undertaken at the multicellular organism level. This will help to establish links between condensate structure and physiological roles. Secondly, current research primarily focuses on the protein and RNA components within condensates. However, other biomolecules, such as metabolites, ions, and chemicals, can also be recruited or stored in condensates in both physiological and pathological contexts (Klein et al. 2020; Dumelie 2023). Therefore, techniques should be developed or adapted to discover the metabolome, ions, and chemical identities of condensates, ideally without disturbing their integrity. The ways in which these biomolecules modulate condensate properties should also be addressed. Thirdly, the components of condensates are context dependent (Cui, et al. 2023; Markmiller, et al. 2018). Dissecting the components of condensates under different stress conditions, at various developmental stages, and in normal versus pathological conditions, will reveal new regulatory mechanisms of condensates.

**Table 2:** Identification of novel biomolecular condensates and biomolecular condensates components

	**Technology**	**Principle**	**Application**	**Advantage**	**Shortcoming**
Identification of novel biomolecular condensates	b-isox precipitation	The microcrystal surface of b-isox act as an organized template, stimulating the transformation of low-complexity sequences from a soluble state to polymerized, fiber-like structures	Globally screen for proteins with the potential for phase separation	1. A resource of candidate proteins with phase separation potential can be readily generated 2. Suitable for protein sample from all species	1.Many proteins with low expression levels are difficult to capture 2. Cannot capture protein that phase separation via multivalent interactions between well-folded domains
	Heterogeneous expression of target proteins fused with fluorescent proteins	Candidate proteins are fused with fluorescent proteins and expressed transiently or stably in cell lines or yeast systems	Screen for candidate proteins with the potential for phase separation	1. Candidate proteins with the potential for phase separation can be readily identified 2. The expression system utilized is genetically and molecularly tractable	1.The overexpression of candidate proteins may introduce artifacts 2. The choice of fluorescent tags can influence phase-separation dynamics and protein function
	Bioinformatics based approach	Compute proteins' phase separation propensity based on the characteristics of amino acid residues and experimental data	Predict proteins bioinformatically with phase separation propensity	1. Only protein sequences or identities are required for computation 2. A vast pool of candidate proteins with phase separation potential can be readily generated	1. Predicted proteins require further experimental confirmation 2.Protein post-translational modifications cannot be used as predictive factors 3. More phase separation features are needed to improve prediction accuracy
Identification of components of biomolecular condensates	Immunoprecipitation	Immunoprecipitate marker proteins within biomolecular condensates	Isolate biomolecular condensates using antibodies against marker proteins	Readily purify biomolecular condensates	1.Weak or transient interactions are often lost; likely only core structure is purified 2. Only a few condensates such as stress granules are purified using this approach
	FACS sorting	Purify specific biomolecular condensates using fluorescent protein-based flow cytometry	Isolate biomolecular condensates with FACS	Efficiently enrich biomolecular condensates to facilitate protein and RNA components identification	Only a few condensates, such as P-bodies, have been isolated using this method, which may not be widely applicable to other biomolecular condensates
	Proximity labeling	Fuse a genetically encoded biotin ligase to marker proteins for proximity labeling of components	Identification of protein and RNA components within biomolecular condensates	The protein and RNA components can be biotin-labeled prior to cell lysis, ensuring no loss of components during the enrichment process	1. The workflow of APEX2 maybe cytotoxic 2. Biotin ligases may influence the function of fused protein and the formation of condensates

**Table 3:** Study the properties and assembly principle of biomolecular condensates

Technology	Principle	Application	Advantage	Shortcoming
In vitro reconstitution	Assemble a fine model of biomolecular condensates outside the complicating environment of cells using purified proteins	Reconstitution biomolecular condensates in vitro to study their properties and assembly principles	1. The biological function of the entire condensates can be verified in vitro 2. Easy to study core component's properties under many different conditions	1. In vitro results alone are insufficient, as they lack actual biological or physiological relevance 2. The concentrations of proteins and nucleic acids used may exceed physiological levels
In vivo reconstitution	Fusion of chemical- or light-controlled dimerization or oligomerization domains to IDRs	Spatiotemporal control phase separation within cellular contexts	1. The properties of constructed condensates are closer to the natural state 2. Active control protein phase separation in vivo	Induced condensates under repeated or strong induction conditions may lead to toxicity
1,6-Hexanediol (1,6-HD)	Disrupting the weak hydrophobic intermolecular interactions	Study the physical state of condensates and the biological function upon the loss of condensates	1. Suitable for in vivo and in vitro studies 2. Sufficiently weak to selectively disrupt liquid like biomolecular condensates while leaving solid-like assemblies and membrane-bounded organelles unaffected	The concentration and duration of 1,6-HD treatment may induce cellular apoptosis and secondary effects
Fluorescence recovery after photobleaching (FRAP)	Study the dynamics of fluorescently labeled biomolecular condensates within living cells by selectively bleaching a small region and observing the subsequent recovery of fluorescence over time	Study the physical state of biomolecular condensates (Liquid, gel and solid)	1. Enables quantitative measurement of the kinetics of phase separation 2. high spatial resolution, allowing selective photobleaching of specific regions within phase-separated condensates	1. The process of photobleaching can introduce cellular stress and perturb the local environment, potentially affecting the behavior of the biomolecular condensates under study 2.The high-intensity light used for photobleaching can cause phototoxicity, leading to cellular damage
Microscope	Optical principles to observe, image and analyze biomolecular condensates	Visualize and analyze the spatial distribution of biomolecular condensates; Reveal substructures of biomolecular condensates	1. The most direct methods to observe phase separation 2. The spatiotemporal behavior of biomolecular condensates can be imaged and analyzed	1.Photobleaching can be induced during imaging 2.Fluorescent proteins can alter the behavior or property of biomolecular condensates
Fluorescence resonance energy transfer (FRET)	The non-radiative transfer of energy from a donor fluorophore to an acceptor fluorophore when they are in close proximity (typically within 1–10 nm)	Detecting interactions between fluorescently labeled molecules within phase-separated condensates	1. Highly sensitive to changes in the distance between the donor and acceptor fluorophores 2. Simultaneously study multiple components or molecular interactions within phase-separated systems	1. Require meticulous experimental design and data analysis 2. Limit by the spatial resolution of optical microscopy 3. Interpreting FRET data in the context of phase separation can be complex due to the influence of factors such as molecular crowding, heterogeneity, and dynamic changes in phase-separated domains

## Data Availability

Not applicable.
